# Uterine Carcinosarcomas (Malignant Mixed Müllerian Tumours): A Review with Special Emphasis on the Controversies in Management

**DOI:** 10.1155/2011/470795

**Published:** 2011-10-05

**Authors:** Rani Kanthan, Jenna-Lynn Senger

**Affiliations:** ^1^Department of Pathology and Laboratory Medicine, University of Saskatchewan, Saskatoon, SK, Canada S7N 0W8; ^2^Room 2868 G-Wing, Royal University Hospital, 103 Hospital Drive, Saskatoon, SK, Canada S7N 0W8

## Abstract

Uterine carcinosarcomas (MMMT—malignant mixed Müllerian tumours) are highly aggressive, rare, biphasic tumours composed of epithelial and mesenchymal elements believed to arise from a monoclonal origin. While hysterectomy with bilateral salpingo-oophorectomy remains the mainstay treatment, high rates of recurrence and metastases suggest a need for lymphadenectomy and postoperative adjuvant treatment. There are no established consensus guidelines for therapeutic patient management. Though well recognized that it improves locoregional control, the role of radiation in improving overall survival outcomes remains undecided. Although various combinations of chemotherapy have been explored, an optimal therapeutic modality is yet to be determined. As overall survival rates have not improved in thirty years, it is suggested that targeted chemotherapy and/or a multimodality approach may yield better outcomes. This paper provides a summary of the aetiopathogenesis of carcinosarcomas (MMMT) limited to the uterus with special emphasis on the controversies in the management of these patients.

## 1. Embryology and Historical Perspectives

The name “malignant mixed Müllerian tumor” (MMMT) is derived from observations of the embryonic female genitalia. During the sixth week of embryogenesis, the Müllerian (paramesonephric) ducts created from intermediate mesoderm of the coelomic epithelium invaginate lateral to the mesonephric ducts. Epithelial and mesenchymal structures arise or are induced from the development of these Müllerian ducts [1]. In males, anti-Müllerian hormone secreted by the Sertoli cells of the testis causes rapid regression of these ducts; however, in females, this duct leads to the formation of the fallopian tubes, uterus, cervix, and cranial portion of the vagina. Certain Müllerian-type carcinomas have been identified, and metaplastic transformation of these carcinomas into sarcoma has been suggested on the basis of clonality analysis [2]. This is further supported by the finding that aside from the uterus, MMMTs have been identified, in decreasing order of frequency in the vagina [3], cervix [4], ovary [5], and most rarely the fallopian tube [6]. Additionally, on rare occasions, the female peritoneum can develop Müllerian-type neoplasms including MMMT [2]. 

For over 150 years, malignant neoplasms arising in the uterus composed of both epithelial and mesenchymal elements have been a subject of debate. Its origin dates back to 1852, wherein it was recognized as a mixed mesodermal tumour that was then called “enchondroma” [1]. Traditionally, MMMTs were thought to be primarily sarcomatous, and therefore, clinical trials and advances in treatment protocols followed this guideline. This assumption has since changed, with the carcinomatous component being favoured as the primary determinant of tumour aggressiveness resulting in a change in the management styles. 

Our current understanding is that an MMMT is a biphasic tumour of the female genital tract, composed of epithelial and mesenchymal tissues. Alternative names in the literature include “malignant mesodermal mixed tumour,” “metaplastic carcinoma,” and “carcinosarcoma” [7]. The nomenclature presently in vogue in North America is “carcinosarcoma” rather than MMMT, and therefore, “uterine carcinosarcoma” is used for this tumour in the remainder of the paper. Based on their sarcomatous component, two categories of uterine carcinosarcomas have been identified: homologous and heterologous. The homologous-type has a sarcoma composed of tissues native to the uterus such as endometrium or smooth muscle whereas in the heterologous-type cartilage, skeletal muscle, or bone is present which is not native to the uterus.

## 2. Materials and Methods

Using PubMed and Google Scholar, a literature search was performed using the text phrases “Malignant Mixed Müllerian Tumor,” “MMMT,” and “uterine carcinosarcoma” limited to review articles in English published in the last ten years (2000–present). Articles were additionally restricted to carcinosarcomas of the uterus with exclusion of those describing this tumour arising elsewhere. The PubMed “Related Articles” feature identified additional relevant articles. The reference lists from these retrieved papers were analyzed to identify additional relevant publications. This process was then repeated twice: (a) with the same key words to identify all papers (case reports, series, and studies) conducted in the past two years (2009–2011) in order to report the most up-to-date findings and (b) with the same key words in combination with “MRI,” “CT,” and “PET” without the date constrictions due to a paucity of material retrieved initially. All relevant publications were collected and reviewed. In total, 74 documents were analyzed in detail and the findings are summarized in this paper.

From the collected bank of references, all studies conducted in the past three years (2008–2011) with *n* > 500 were selected for in-depth review. Six papers [8–13] were identified. Collectively consisting of 13,388 patients, the demographics and treatment modalities of these major studies are analyzed in detail and discussed in this paper.

## 3. Epidemiology

**Figure 1:**
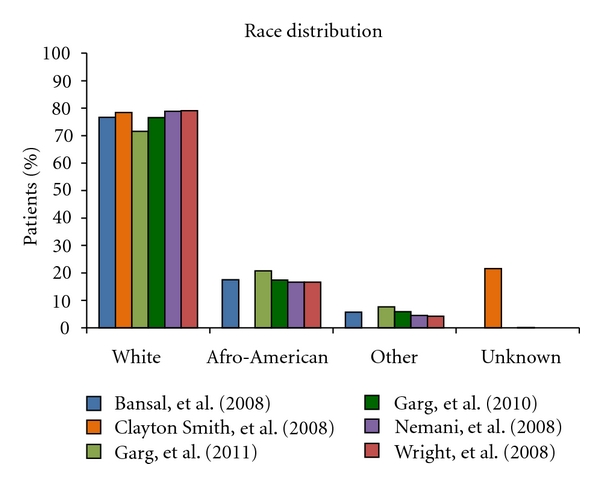
This column-graph demonstrates the race distribution among the six chosen index large case-based series of Bansal et al. [8], Clayton Smith etal. [9], Garg et al. [10], Garg et al. [11], Nemani et al. [12], and Wright et al. [13]. The *y*-axis indicates the percentage of patients in the respective study that fall into each category. Contradictory to the commonly held belief that Afro-Americans are more likely to develop uterine carcinosarcoma than Caucasians, all six studies had a white population much greater than the Afro-Americans.

**Figure 2:**
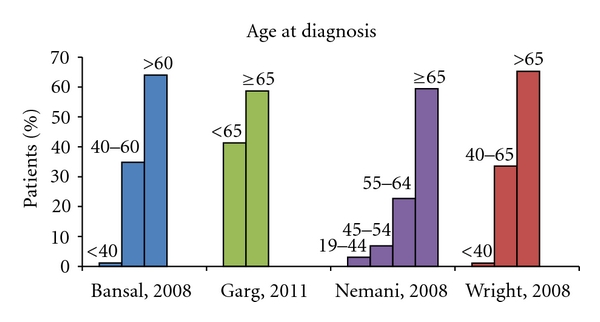
This column graph demonstrates the age distribution among the four of the six index large case-based series of Bansal et al. [8], Garg et al. [10], Nemani et al. [12], and Wright et al. [13]. Garg et al. [11] and Clayton Smith etal. [9] are not included, as this data was not provided. The numbers on top of each bar indicates the age range it comprises, as each study categorized patients within different age groupings. This graph demonstrates the predominance uterine carcinosarcomas have for an older, postmenopausal population.

Carcinosarcomas though rare, representing less than 5% of all uterine tumors [2], account for 16.4% of all deaths caused by a uterine malignancy [14]. The age-adjusted rate of uterine carcinosarcoma is reported at 0.6/100,000 [11]. Incidence of women over 35 years of age affected by carcinosarcoma is 1.8 white and 4.3 black women per 100,000 in the United States [15]. Afro-American women are at a greater risk of developing carcinosarcomas when compared with Caucasians, at a 2.2 to 3.0 ratio [11] and thus carcinosarcoma patients are more often nonwhite (23% versus 15%) [16]. However, detailed comprehensive analysis of the six indexed large case-based studies [8–13] show trends that do not support this traditionally held race distribution pattern (Figure 1). Women are usually over the age of 50, with most cases occurring between the sixth and seventh decade [17], with a median age of 62 years [18] as demonstrated in Figure 2. 

Risk factors for the development of carcinosarcoma are similar to those of endometrial carcinoma and include nulliparity, advanced age, obesity, exposure to exogenous estrogens, and long-term use of tamoxifen [19, 20]. Tamoxifen is associated with a 2–7x greater risk of developing endometrial malignancies. Specifically, carcinosarcomas have been reported to occur 7–20 (median of 9 years) years after the initiation of this regime [21]. On the contrary, oral contraceptives are reported to provide a protective effect against these tumours [16].

## 4. Aetiology

Carcinosarcomas are composed of two histological subtypes which are classified based on the appearance of the sarcomatous component. The sarcoma of heterologous type has been described as rhabdomyosarcoma, chondrosarcoma, osteosarcoma, or liposarcoma, whereas the homologous type tends to be fibrosarcoma, endometrial stromal sarcoma, or leiomyosarcoma. In both cases, the carcinomatous component may be composed of endometrioid, serous, or clear cell type [22]. Aetiological factors implicated in the development of this cancer include pelvic exposure to irradiation, obesity, nulliparity, and exposure to the human papilloma virus or exogenous estrogen [23]. Identification of these two individual components of carcinosarcomas has sparked theorization to their origin, of which three predominant theories are proposed [19, 22].

1. The *collision theory* suggests that the two components had separate points of origin prior to their “colliding” together to form a single tumour.
2. The *combination theory* postulates that a common stem cell precursor undergoes bidirectional differentiation that results in the creation of the two histological types.
3. In *conversion theory,* a single epithelial component is hypothesized to undergo metaplastic differentiation from which the mesenchymal component is derived.

It is currently believed that carcinosarcomas have a monoclonal origin from a common multidirectional progenitor stem cell. Though epithelial markers are expressed in more than 60% of the sarcomatous component, mesenchymal marker expression is rare in the carcinomatous element [1]. Clinical, pathological, and molecular observations suggest that these neoplasms are derived from the Müllerian epithelium's single stem cells, with metaplasia or dedifferentiation resulting in the sarcomatous elements [1]. Such a monoclonal origin may be explained by both the combination and conversion theories [7]. Cell cultures, ultrastructural studies, and immunohistochemical analyses all support the conversion theory for the tumorigenesis of this neoplasm [22]. Traditionally, carcinosarcomas were classified as sarcomatous; however, recent evidence suggests that the epithelium may be the principle “driving” component. The histogenesis still remains poorly defined [14]. 

Despite the majority of reports supporting the conversion theory, there remains a percentage of carcinosarcomas with a biclonal origin [7]. Though 70%–80% of staining with p53 is identical between the sarcomatous and the carcinomatous components, 10%–15% of cases have distinctive morphologies, suggesting different origins [24]. It is, therefore, suggested only a small subset of carcinosarcomas may be “true” collision tumours [25]. 

Carcinosarcomas may be a radiation-inducible tumour. Twenty years ago, it was reported that pelvic irradiation may be implicated in the development of extremely aggressive uterine cancers, particularly sarcomas. At this point, it was noted that in one study, five of the eight patients with uterine malignancies had a previous pelvic malignancy treated by radiation [26]. It is now estimated that 5%–30% of patients with carcinosarcoma have a history of pelvic irradiation. These neoplasms will often be diagnosed after a latent period of 14 years after irradiation [27]. A recent study by Callister et al. found that 11% patients diagnosed with carcinosarcoma had a history of prior pelvic radiation therapy, 17 for malignant, and 15 for benign disease, which negatively influenced pelvic control[28].

## 5. Clinical Features

The clinical presentation of carcinosarcomas may be nonspecific, with symptoms that are similar to other pelvic neoplasms [29]. A typical presentation of carcinosarcoma includes pyometra with vaginal bleeding, bloody or watery discharge, abdominal pain, or as a polypoid mass in an older, postmenopausal woman, as listed in Table 1 [7]. At physical exam, 50%–95% of patients have enlargement of the uterus with 50% of patients having protrusion of a polypoid lesion through the endocervical canal [23]. The “symptom triad” indicative of carcinosarcoma rather than endometrial adenocarcinoma includes pain, severe vaginal bleeding, and the passage of necrotic tissue per vaginum [27]. Additionally, patients may be asymptomatic or present with anemia [15]. Patients are often overweight and hypertensive and may be diabetic or have poor performance status [30]. Aside from the physical exam, routine pretreatment assessments also include blood work and chest X-rays, as well as pyelography, cystoscopy, proctoscopy, and bone scans if required [31]. Elevated levels of serum CA-125 have also been reported with this neoplasm [32].

The pathological staging and histological features of the carcinomatous component of carcinosarcoma are responsible for the tumour's biological potential and aggressiveness. Increased aggressiveness is associated with atypical carcinosarcomas with unusual neuroendocrine or melanocytic differentiations [33]. Over half (53%) of carcinosarcoma patients present with advanced-stage disease [16]. Of patients with localized carcinosarcoma, 20% will be upstaged at laparotomy due to the presence of regional lymph node metastases [34]. A simple working classification for the staging of carcinosarcoma tumours is as folows: stage I tumours are confined to the corpus uteri, stage II tumours involves both the corpus and the cervix, stage III tumours are limited the lesser pelvis, and stage IV tumours have extrapelvic extension.

## 6. Pathology

### 6.1. Gross Features

Uterine carcinosarcoma's gross histological appearance is usually that of a solitary polypoid mass with regions of haemorrhage and necrosis projecting into the uterine cavity [35]. Gritty or hardened areas may suggest osseous or cartilaginous differentiation [16]. In 50% of patients, a polypoid mass within the endocervical canal is present [23]. Within the uterus, carcinosarcomas most commonly arise on posterior wall of uterine body near the fundus [29]. The mass is generally large and soft, and grows to fill and distend the uterus [23]. Due to increased cellularity and sarcomatous differentiation, tumours may be bulkier, fleshier, and larger than endometrial adenocarcinomas [16, 36]. Advanced disease at clinical presentation is found in approximately 60% of patients, with gross evidence of tumour extension beyond the uterus [7].

### 6.2. Microscopic Features

Carcinosarcomas are characterized by their unique biphasic morphology, a tumour composed of both epithelial and mesenchymal elements. Microscopically, these two elements may be intermittently mixed or be seen as two distinct components [37]. The epithelial component is often a high-grade carcinoma such as papillary serous (66%) or endometrioid (42%) [7] though it may be composed of a variety of histological subtypes including squamous cell carcinoma, basaloid squamous carcinoma, adenocarcinoma, adenosquamous carcinoma, adenobasal carcinoma, adenocystic carcinoma, or an undifferentiated carcinoma [3]. Unlike conventional adenocarcinomas, solid areas of marked pleomorphism, bizarre cells, embryonal glandular growth patterns and lace-like arrangement of cells may be present [16]. The mesenchymal element may be (a) homologous, containing cells native to the uterus including stromal sarcoma, fibrosarcoma, undifferentiated sarcoma, or leiomyosarcoma (2%) or (b) heterologous with mixed components including rhabdomyosarcoma (18%), chondrosarcoma (10%), osteosarcoma (5%), or liposarcoma (1%). One-third of carcinosarcomas have two or more sarcomatous elements, with high-grade stromal sarcoma being the most common type [7]. Choriocarcinoma and melanocytic differentiation are unusual [33, 37].

### 6.3. Immunohistochemical Features

Carcinosarcomas express epithelial (epithelial membrane antigen (EMA), pancytokeratin) and stromal lineage markers in relation to their histological appearances such as desmin in muscle differentiation or S100 in areas with chondroid or lipomatous differentiation. A number of studies have attempted to evaluate the differences of protein expression between the two components as prognostic/predictive markers, however, often resulting in inconclusive results. This perhaps is attributed to (a) rarity of this neoplasm, (b) small sample size of case series, (c) tumour heterogeneity, and (d) variations in methodology limiting comparative analysis. 

Besides the study of lineage immunohistochemical markers to establish aetiopathogenesis in carcinosarcomas, there are a number of case studies and reports on cell cycle proliferative markers and apoptotic regulatory proteins that explore the possibility of identifying molecular profiles as potential therapeutic targets or markers of prognosis [24, 38]. Overexpression of tyrosine kinase receptors such as HER-2, EGFR, and KIT suggest potential targets for therapeutic use in subgroups of carcinosarcoma [32, 39–42].

## 7. Radiology

Traditionally, diagnosis of carcinosarcoma is most often made postoperatively by histological examination and immunohistochemical studies. Current research is aimed at determining preoperative imaging criteria to differentiate this tumour type from other uterine malignancies, particularly endometrial carcinomas due to the differences in treatment and prognosis. Preoperative diagnosis of uterine carcinosarcoma will facilitate the planning of appropriate surgical management with adjuvant therapy. 

### 7.1. Magnetic Resonance Imaging (MRI)

Initial characterizations of uterine carcinosarcoma by MRI as outlined by Worthington (*n* = 4) in 1986 described carcinosarcoma as a large mass in the pelvis that entirely obliterated the architecture of the uterus, with inhomogeneously low intensity of T1W1 and a heterogeneous appearance on T2W1 [15, 43]. These findings were further supported in 1980 when imaging studies by Shapeero and Hricak (*n* = 7) documented deep tumour invasion of the myometrium [44]. Current literature disagrees with these findings, concluding that most carcinosarcomas are visualized as exophytic lesions with no evidence of invasive growth. This discrepancy may be partially due to different clinical stages of the lesions examined or because of increasing spatial resolution of MR images over the past twenty years allowing for better distinction of the border between the tumour and the myometrium [15]. 

More recent studies report most of these tumours to be sharply demarcated [44] with endometrial cavity distension. In the recent study by Bharwani et al. in 2010, one of the largest series to study MRI characteristics (*n* = 51) 76% of tumours were well defined with 61% having irregular margins. Only 12% were reported as aggressive with architectural destruction. On T1-weighted images, the majority of uterine carcinosarcomas were isointense to the myometrium (76%) and the endometrium (71%) compared with endometrial carcinoma that was isointense to both these elements in 59% of cases. T2-weighted images found hyperintensity of uterine carcinosarcomas to the myometrium (92%) and hypointensity (55%) or isointensity (41%) to the endometrium, a finding that is highly comparable to endometrial carcinoma (97% hyperintense to myometrium, 23% isointense, and 68% hypointense to endometrium). The craniocaudal dimension of uterine carcinosarcoma was larger than endometrial carcinoma. This study found 88% of uterine carcinosarcomas to be indistinguishable from endometrial carcinoma on MRI. There was no significant difference in the extent of myometrial invasion between these two lesions [45]. These results support the findings of the 2008 investigation by Tanaka et al. (*n* = 17) that reported uterine carcinosarcomas to be large exophytic tumours with minimal uterine architectural destruction [15]. 

Though on MRI uterine carcinosarcomas may be indistinguishable from endometrial carcinomas, their poor prognosis necessitates radiologists to consider them in the differential diagnosis of strongly enhanced uterine lesions [15]. Enhancement equal to or greater than that of the myometrium suggests the possibility of this tumour-type [45]. Clinicopathological correlation with MR images is often necessary to accurately diagnose these rare tumours preoperatively [36].

### 7.2. Computed Tomography (CT)

Imaging of uterine carcinosarcoma by CT scans is not as well-described as MRI studies. The appearance of uterine carcinosarcoma is not pathognomonic and can be easily mistaken for lesions such as leiomyosarcomas or endometrial carcinomas [46]. Dilatation of the uterus is a common finding reported in 90% and 73% of patients in two studies [46, 47]. In one study, myometrial invasion was evident in 80% of patients, detected by contrast-enhanced computed tomography (CECT) by the differences in attenuation between the tumour and the myometrium. It was further recognized that CECT has potential for tumour staging, with a reported accuracy of 89%. This modality shows the tumour to be a heterogenous, hypodense, ill-defined mass [47].

### 7.3. Transvaginal and Transabdominal Sonography

Sonography is a noneffective investigation for uterine carcinosarcoma. Doppler imaging may be unable to (a) accurately predict tumour stage, (b) evaluate the retroperitoneum, and (c) evaluate the deep pelvic lymph node chains [47]. Though most Doppler ultrasonography is able to detect areas of neovascularization associated with malignant tumours, it was not able to detect the hypervascularity of a uterine carcinosarcoma in a reported case [48]. These tumours are inhomogeneously echoic, with small cystic spaces that are anechoic [47].

### 7.4. ^18^F-Fluorodeoxyglucose Positron Emission Tomography (^18^F-PDG PET) Scans

Though scarcely reported in the literature, ^18^F-PDG PET scans show potential in the detection of metastases from uterine carcinosarcomas. Malignant tissue has a greater rate of glucose metabolism than benign; therefore, suspected malignancies and their metastases can be visualized [48]. One study researching four uterine sarcomas and one uterine carcinosarcoma found ^18^F-FDG PET was able to accurately predict all five primary malignant tumours, whereas MRI predicted four of them, and ultrasound only two [49]. ^18^F-PDG PET scan use by Ho et al. allowed for 36.8% of the patients in their study to be re-evaluated, two-thirds for monitoring response and one-ninth to be restaged. Though this modality appears to offer the possibility of earlier detection of metastases, there was no reported improvement in patient outcome data in this study [31]. Another study by Murakami et al. suggests that in patients with recurrent uterine carcinosarcomas, FDG-PET may increase prolonged survival, especially in those with small tumours treated with combination therapy [50]. ^18^F-PDG PET scans seem to have limited value in posttherapy surveillance or restaging after failure with recurrence [31].

## 8. Treatment

To date, no national consensus guidelines have been established for the management of uterine carcinosarcomas [36]. The optimal treatment remains uncertain, partially because the histogenesis remains controversial [5]. Therapeutic approaches may differ depending on the precursor lesion [14]. Chemotherapy effectiveness in sarcomas differs greatly from that in endometrial carcinomas, with increased toxicity [51]. A full understanding of the pathobiogenesis of this tumour is necessary to predict the “gold standard” treatment. As it is currently believed that uterine carcinosarcoma is akin to a metaplastic endometrial carcinoma, most treatment plans have been modeled based on treatment protocols for high-risk endometrial carcinoma [19]. 

The primary treatment option remains surgery; however, high rates of relapse and metastases postoperatively necessitate effective adjuvant therapies [28]. As research continues to elucidate the natural history of uterine carcinosarcomas, with recognition of the high rates of recurrence and distant metastases, it is proposed by some authors that systemic chemotherapy should replace radiotherapy as the primary modality of adjuvant treatment [52]. Regardless, in higher-staged tumours, neither radiotherapy nor chemotherapy provides any significant overall survival benefit [53] and there remains to date no consensus to guide therapeutic strategies for the various stages of disease [14]. 

Despite advances in adjuvant therapy, the past four decades have not seen any measurable improvement in survival. It is, therefore, suggested that the primary curative treatment is surgical resection [28]. A multimodality treatment plan has been suggested, with results indicating that surgery followed by a combination of both chemotherapy and radiation therapy yields a significantly longer median disease-specific survival (DSS) of 31 months versus surgery alone (DSS = 3 months), radiation therapy alone (DSS = 15 months), or chemotherapy alone (DSS = 14 months) [14]. These findings are further supported by a study by Menczer et al. demonstrating that uterine carcinosarcoma patients undergoing sequential treatment of chemotherapy and irradiation not only have less toxic events, but also have a 50% and 80% decreased mortality compared to patients taking irradiation and chemotherapy alone [54]. 

### 8.1. Surgery

Although total abdominal hysterectomy (TAH) with bilateral salpingo-oophorectomy (BSO) is the preferred standard surgical option, the additive benefit for the role of lymphadenectomy remains undetermined [55]. The current surgical practice recommended for uterine carcinosarcoma is surgical staging with TAH with BSO, pelvic lymphadenectomy, and para-aortic lymph-node sampling with peritoneal washings. The role of pelvic and para-aortic lymph-node sampling, the method, technique of dissection, and the optimal number of lymph nodes to be sampled remains undetermined [12]. For patients with advanced disease, cytoreduction surgery is recommended based on their previous experiences with ovarian and other uterine neoplasms [7, 56]. In 2010, Garg et al. studied this relationship and found that the risk of death decreased 33% in patients that underwent a lymphadenectomy when compared to those that did not [11]. These results are similar to Nemani's results, that reported a median survival of 54 months in patients who underwent a lymphadenectomy (5-year overall survival of 49%) compared to 25 months in those that did not (5-year overall survival of 34%) [12]. Other studies have found the addition of lymphadenectomy to be an independent positive prognostic factor [8, 10].

**Figure 3:**
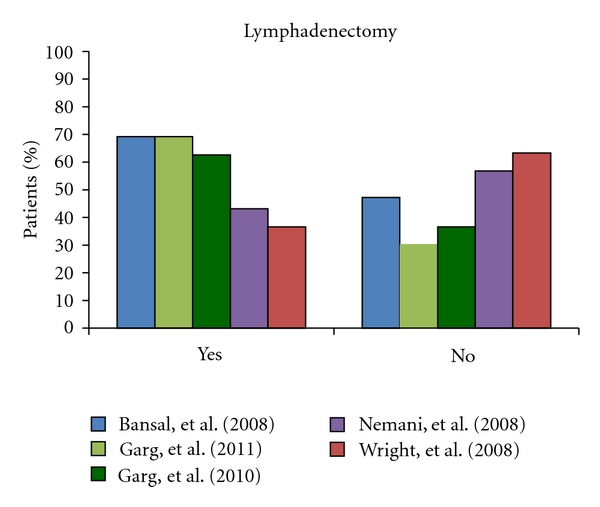
This column graph presents the percentage of patients who underwent lymphadenectomy among five of the six chosen index large case-based series of Bansal et al.[8], Garg et al. [10], Garg et al. [11], Nemani et al. [12], and Wright et al. [13]. Clayton Smith et al. [9] is not included as this data was not provided. This graph demonstrates that despite convincing evidence indicating the importance of lymphadenectomy as part of surgical treatment of uterine carcinosarcomas, a substantial proportion of patients do not undergo lymph-node dissection.

Three primary arguments in support of conducting a lymphadenectomy in all patients with uterine carcinosarcoma have been put forward, including (a) accurate staging will allow the determination of the patient's true “metastatic risk”, (b) possible reduction in locoregional recurrences within the lymph nodes, and (c) improving selection of patients for adjuvant therapy. Lymphadenectomy offers a survival advantage only for node-negative patients, as removal of positive nodes upstages the disease and worsens the prognosis. By contrast, “negative nodes” may contain micrometastatic foci that, when removed, does decrease the risk of the development of macrometastases [55]. In Nemani's study, 14% patients had positive nodes at lymphadenectomy. Node-negative patients may then be referred for adjuvant therapy. Prognosis is significantly improved in patients who receive both lymphadenectomy and adjuvant radiotherapy when compared with those who were treated by hysterectomy and bilateral salpingo-oophorectomy alone [12]. In 2010, Vorgias and Fotiou reviewed the uterine carcinosarcoma literature and found that between 35% and 57% of uterine carcinosarcoma surgeries carry out lymph node dissection though the extent ranges from biopsy to complete pelvic lymphadenectomy [55]. The number of nodes removed has been reported to have no significant impact on overall survival by some authors [12]; however, others have found that in early-stage uterine carcinosarcoma, the number of nodes removed is a risk factor correlated with both recurrence and survival [34]. Congruent with these findings, a recent publication by Garg et al. in 2011 concludes that the optimal patient management for uterine carcinosarcomas includes abdominal hysterectomy, bilateral salpingo-oophorectomy, lymph-node dissection, resection of gross abdominal disease, and sampling of peritoneal washings [10]. Despite this conclusion, composite data analysis of the six large index case series as seen in Figure 3 illustrate that a substantial percentage of patients are still not receiving any lymph-node dissection either synchronously or metachronously in conjunction with their TAH + BSO.

### 8.2. Radiotherapy

**Figure 4:**
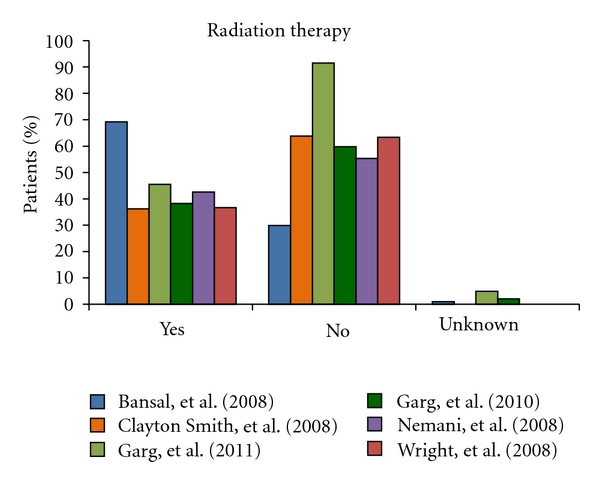
This column-graph shows the percentage of patients that underwent radiation therapy in each of the six chosen index large case-based series of Bansal et al. [8], Clayton Smith etal. [9], Garg et al. [10], Garg et al. [11], Nemani et al. [12], and Wright et al. [13]. Though in five of the six studies more patients did not have radiation therapy as part of their treatment regime, the difference is not significant. This is probably best explained by the remaining unanswered controversy that questions the improvement in survival rates associated with this modality of adjuvant therapy.

It is well established that radiotherapy contributes to decreased pelvic recurrences; however, the impact this adjuvant postoperative therapy has on patient survival remains a subject of controversy. Data describing the relationship between survival and uterine carcinosarcomas is limited [55]. Recognition of the high levels of recurrence and metastatic spread associated with uterine carcinosarcomas has called for a re-evaluation of the role of adjuvant radiotherapy in patient management. Due to small sample sizes, limited surgical staging data and lack of stratification of prognostic factors, it is difficult to make conclusions based on the current literature [57]. As seen in Figure 4, though patients are more likely not to receive radiotherapy, the differences amongst the large case-based series are not significant.

Some studies have found pelvic irradiation yielded only slight improvement in pelvic recurrence rate presumably because of the increased tendency for intraperitoneal reseeding. Though radiation therapy may improve locoregional control, demonstration of a survival advantage remains uncertain [10]. Callister et al. (*n* = 300) associated adjuvant radiation therapy with lowered pelvic recurrence rate and a decreased time interval to distant metastatic spread; however, no statistically significant overall survival benefit was found [28]. Sartori (*n* = 118) additionally found no improvement in 5-year disease-free survival (DFS) in patients receiving postoperative radiation [58]. It is suggested that the inability of studies to show statistically significant overall survival (OS) rates in patients receiving adjuvant radiotherapy may be due to the difference between clinical and surgical staging, as 9% of patients with “early clinical stage” will be upstaged to stage III and 10% to stage IB because of metastases, thus diminishing the possible long-term survival effects of radiotherapy. It is also suggested that by extending the field of radiation to include the abdomen and the regional lymph nodes, patients who are upstaged may receive some benefit from this technique [57]. 

In contrast, other studies have demonstrated a prolonged DFS in patients with early-stage disease treated with adjuvant radiotherapy [1]. In a study by Clayton Smith et al. (*n* = 300), radiation therapy increased 5-year survival rates from 33.1% (patients not receiving adjuvant radiation therapy) to 42.4% (patients receiving adjuvant therapy. Multivariant analysis further reported adjuvant radiation therapy conferred benefits for both overall and uterine-specific survival in women stages I–IV, with the greatest impact on Stage IV disease [9]. The benefits of radiation therapy were further elucidated by Nemani et al. (*n* = 1697) who demonstrated a median survival increase from 23 months to 29 months in patients who had not undergone lymph-node dissection with a 5-year OS increase from 33.4% to 35.8% [12]. These findings were supported the same year by Wright et al. (*n* = 1819) who also found that in patients with no history of lymphadenectomy, radiation therapy reduced mortality rates by 25% [13]. In patients with early-stage uterine carcinosarcomas, rates of pelvic recurrence when treated with modern radiotherapy techniques do not exceed 10% [57]. Controversies still remain regarding the techniques of radiation: localized pelvic radiation by vaginal brachytherapy versus whole abdominal radiation by external beam [59].

### 8.3. Chemotherapy

Despite surgical extirpation of the primary tumour, sites of failure occur in both pelvic and extrapelvic regions. Pelvic radiation does not eliminate pelvic relapse. Extrapelvic recurrence/relapse is common with hematogenous, transcoelomic, and lymphatic spread of the tumour; therefore, chemotherapy has a definitive role to minimize both local and distal failure [60, 61]. Identification of effective chemotherapeutic agents to treat patients with uterine carcinosarcomas is essential due to such high incidence of disseminated disease at presentation. In light of the continuing sarcomatous versus carcinomatous debate, traditional adjuvant chemotherapeutic regimes have been created based on the model employed for high-grade sarcomas such as leiomyosarcoma and undifferentiated uterine sarcoma [25]. Chemotherapy response rate in patients with a predominant carcinomatous element yielded a better overall response rate (87.5%) than those with a dominant sarcoma [56]. There is no universal agreement on a postoperative chemotherapeutic regime for uterine carcinosarcomas [7]. Most studies focus on the development of postoperative adjuvant treatment for Stage I/II lesions and palliative treatment for advanced [18]. Active single cytotoxic antineoplastic agents include ifosfamide (RR = 29%–36%), cisplatin (RR = 28%–42%), doxorubicin (RR = 10%–25%), and paclitaxel (18%) [62]. Response rates (RR) to cisplatin are 19% as a first-line and 18% as a second-line agent against uterine carcinosarcomas. RR to paclitaxel is 18% with 4-month duration [63]. Certain single chemotherapeutic agents of note proposed since 2005 are herein summarized.

**(i) Sorafenib [64]**

Sorafenib acts by inhibiting wild-type Raf-1, mutant B-Raf and several receptor tyrosine kinases such as vascular endothelial growth factor receptors (VEGFR). Though commonly used to treat renal cell carcinoma and hepatocellular carcinoma, the Ras/Raf/Mek/MAP pathway is suggested to play a role in uterine cancers. In this context, 16 patients with uterine carcinosarcoma were given a median of 28 days of sorafenib cycles. Adverse events (grade 3+) included hypertension (13%), hand-foot syndrome (13%), hypophosphatemia (7%), and hyponatremia (7%). No objective RR was seen, and the median OS was 5.0 months (range 1.4–14.0 months) with a progression-free survival (PFS) of 1.8 months (1.4–3.5 months range).

**(ii) Topotecan [63]**

Topotecan acts as an inhibitor of topoisomerase 1 regularly used for ovarian and small cell lung cancers and active against several sarcomas and gynecologic cancers. In Miller's study, 48 patients with advanced, persistent or recurrent uterine carcinosarcoma were given different dosages of topotecan. Toxicities included neutropenia (73%), leukopenia (29%), and/or thrombocytopenia (21%) with three deaths due to neutropenic sepsis. The total RR was 10%, with response duration of 8.3 months.

**(iii) Imatinib Mesylate (Gleevac) [65]**

Gleevac acts by inhibiting the Bcr-Abl tyrosine kinase, PDGFR, and c-Kit. In Ramondetta's study [17], 45% of uterine carcinosarcomas stained positively for *Abl* and 100% for PDGFR-*β*. This chemotherapeutic drug was tested on a series of 23 women in Huh's study with persistent/recurrent uterine carcinosarcoma, the majority of which had undergone one prior chemotherapy regime. PFS greater to six months only occurred in one patient, with a median PFS of 1.6 months and median survival 4.1 months. Toxicities reported included fatigue, dehydration, anorexia, and genitourinary/renal/lymphatic/metabolic, and/or ocular toxicities.

The value of combination chemotherapy has become increasingly notable in the past decade, with an objective response rate 50% higher than that reported with single cytotoxic chemotherapeutic agents [51]. Nevertheless, no universal agreement on the best combination of these drugs has been established [7]. Similar to carcinomas, uterine carcinosarcomas are often responsive to platinum-based chemotherapies and may be coupled to DNA-alkylating agents with activity against sarcomas [16]. A variety of agents have been tested in combination with platinum-based chemotherapeutic agents, including adriamycin, dacarbazine, and cyclophosphamide [66]. Though the best-studied combination has been ifosfamide and cisplatin, disappointing response rates (18%–44% for single-agent cisplatin and 39% for single-agent ifosfamide in pretreated patients) limited by severe side effects necessitates further study [52]. In patients with high-grade tumours, ifosfamide and cisplatin have been recognized as highly active agents [18]. Combination chemotherapeutic agents of note proposed since 2005 are herein summarized.

**(i) Cisplatin and Ifosfamide**

RR of the combination of these chemotherapeutics (54%) has been shown to be significantly greater than that of ifosfamide therapy alone (36%) [62]. A study led by Sutton et al. found a slight advantage in the median PFS in patients taking this combination when compared to those on ifosfamide alone. Median PFS was 4.0 months with the single-agent treatment and 6.0 months with the combination, yet no statistically significant difference in median survival was found [67]. In patients with recurrent or metastatic disease, this combination has shown to be highly active agents [18]. This combination of chemotherapeutic agents compared positively over complete abdominal/pelvic radiation for all stages of uterine carcinosarcomas although overall survival did not greatly improve [59]. In Sutton's study of 65 early-stage uterine carcinosarcoma patients, he found 24 month PFS and OS at 69% and 82%, and 84-month at 54% and 52%, respectively [18].

**(ii) Cisplatin, Ifosfamide, and Mesna [17]**

Sixteen patients, 10 with primary uterine carcinosarcomas, were treated with this combination, receiving 1–10 cycles of therapy. After the first cycle, two women died from disease progression, and an additional three were taken off the treatment due to toxicity. Of the remaining six women, the mean number of chemotherapeutic cycles was 3.8. All women experienced gastrointestinal toxicity and neutropenia was a major side effect. No complete response occurred and PFS ranged between 2–4 months.

**(iii) Ifosfamide and Paclitaxel [68]**

The advantage of combining ifosfamide with paclitaxel as opposed to ifosfamide as a single agent was explored by Homesley et al. as part of a Gynecologic Oncology Group (GOG) study. A total of 179 women were included, 91 of which were treated with ifosfamide alone and the remaining 88 with ifosfamide combined with paclitaxel and filgrastim. Alopecia and severe sensory neuropathy were most common in the combination group, whereas nonsevere thrombocytopenia was seen more in patients receiving the single agent. The odds of a therapeutic response in patients receiving the combination was 2.21x greater compared to the solitary agent. Significant differences in PFS (5.8 months versus 3.6 months) and OS (13.5 months versus 8.4 months) were additionally noted.

**(iv) Paclitaxel and Carboplatin [52, 62]**

The Gynecologic Oncology Group (GOG) led by Powell reports a series of 46 patients with advanced-stage uterine carcinosarcoma, two-thirds of which were newly diagnosed. The majority of patients had six or more cycles of paclitaxel-carboplatin chemotherapy. It was found to be both active and well tolerated; the range of RRs reported is between 55% and 80%, and the most common toxicities were hematologic, fatigue, and peripheral neuropathy. This drug regime was additionally determined to be nonexpensive, partially because it can be conducted on an outpatient basis rather than the three-day inpatient admission required for ifosfamide therapy. It is suggested that additional biological anticancer therapies may be added onto this regime. An additional study led by Lacour et al. reported 23 patients with advanced/recurrent uterine carcinosarcoma, the majority (69.2%) of which had previously received radiation therapy, and reported a time to progression (TTP) of 9.5 months and an OS of 21.1 months. Similar to the GOG studies, common toxicities included fatigue, neutropenia, and alopecia. There was no significant difference between the survival of patients with and without measurable disease.

**(v) Gemcitabine and Docetaxel [69]**

This combination of chemotherapeutic agents have been used to achieve RRs of 17%-18% in advanced soft-tissue sarcomas; therefore, 28 patients all who had undergone one prior chemotherapeutic regime were given this combination on a weekly schedule to treat recurrent disease. The RR was disappointing at 8.3%, with no complete response, and a partial response was obtained in only two patients. The median PFS was 1.8 months, and median survival was 4.9 months. Toxic effects included myelosuppression, thrombocytopenia, and anaemia.

Additional chemotherapeutic agents that have been evaluated include piperazinedione, etoposide, mitoxantrone, diaziquone, amonafide, aminothiadiazole, and topotecan; however, they did not demonstrate significant results [63]. The effectiveness of chemotherapeutic agents decreases in the treatment of distant metastases [70]. Response rates of recurrent disease are reported at 18%–36% (ifosfamide), 19% (doxorubicin), 18%-19% (cisplatin) and 9%-10% (topotecan) with gemcitabine and docetaxel having a low response rate [7]. Regardless of whether the chemotherapeutic regime employed is a single or combination agent, treatment of uterine carcinosarcoma will likely have more toxic effects than treatment of endometrial adenocarcinomas [51]. 

It has been suggested that the future of uterine carcinosarcoma therapy may lie in identifying biological agents for targeted chemotherapy. Tyrosine kinase inhibitors may be a viable option as *abl* is expressed in up to 45% of uterine carcinosarcomas, *Her-2* in 19% and PDGFR*β* in 100%. Additional potential targets expressed by these tumours include receptors to estrogen, progesterone, vascular endothelial growth factor, cyclooxygenase 2 and epidermal growth factor [52]. It is likely that further understanding of this rare tumour will facilitate the identification of additional potential antineoplastic targets. Elevated CA125 postoperatively confers a 5.7x risk of death [32] and perhaps could aid in early identification of candidates for adjuvant radiation and/or chemotherapeutic treatments. Postoperative multimodal adjuvant therapy with sequential chemotherapy followed by radiotherapy has to date shown no evidence of measurable survival benefit [7].

## 9. Recurrence and Metastases

Recurrences in uterine carcinosarcomas occur in over half of patients after primary surgical and adjuvant therapy [7]. Even in early-stage disease, rates of recurrence are reported between 47%–64% [36] and up to 80% of these will be associated with distant metastases [52]. Specific factors that increase the risk of recurrence include patient age, adnexal spread, metastases to the lymph nodes, tumour size, lymphatic-vascular space involvement, histologic grade, cell type, peritoneal cytologic findings, and the depth of invasion of the primary tumour. Interestingly, on multivariate analysis, only adnexal spread, lymph-node metastases, sarcoma cell type, and sarcomatous grade were positive predictors of recurrence [7]. Most recurrences occur within one year [14]. 

Local recurrences to the pelvis and abdomen are more often the cause of death in patients with uterine carcinosarcoma than metastatic disease. Additionally, the dissemination pattern of uterine carcinosarcoma is unpredictable. Metastatic disease is reported to be related to positivity of the retroperitoneal lymph nodes, deep myometrial invasion, cervical tumour extension, vascular invasion, and a low-degree of differentiation [1]. In contrast to sarcomas that spread haematogenously, uterine carcinosarcomas behave like endometrial carcinoma and spread through the lymphatics. It is not surprising then that the tumor emboli retrieved from both within these lymphovascular channels and the metastatic lesions almost always contain elements of carcinoma with or without a coexisting sarcoma, and solitary sarcomatous metastasis is uncommon [25]. Metastatic uterine carcinosarcoma is usually clinically asymptomatic. The most common sites of metastatic deposit include the lung (49%), peritoneum (44%), pelvic or para-aortic lymph nodes (35%), adrenal gland or bone (19%), heart or pericardium (9%), and/or brain (7%) [31]. Additional sites of metastases include the pancreas, liver, thyroid gland, eye, and skin [53]. Uterine carcinosarcoma has the highest rate of pulmonary metastases among uterine malignancies [45]. Recurrent or metastatic uterine carcinosarcomas are often treated with chemotherapy [51].

## 10. Prognosis

Although uterine carcinosarcomas account for less than 5% of all uterine malignancies, they are responsible for over 15% of uterine cancer-related deaths [7]. Over the past thirty years despite evolving and advancing therapeutic regimes, prognosis remains poor, with no significant improvement in survival or recurrence rates [36]. Stage is reported as an independent prognostic factor for overall survival in patients with uterine carcinosarcoma [30]; however, the comparison of survival data outcome in the published literature is difficult due to (a) lack of stage stratification in major large published series and (b) lack of standardization compared to survival outcome data in endometrial carcinomas or uterine leiomyosarcomas. Nevertheless, higher stage disease is correlated well with decreased overall survival. 5-year survival rates in early uterine carcinosarcomas (FIGO Stages I/II) are between 30%–46%, and 0%–10% in advanced cancers (FIGO Stages III/IV) [19]. The median survival in patients with uterine carcinosarcoma ranges between 16 and 40 months [11] with death usually occurring within 1-2 years of the initial diagnosis [71]. The prognosis of uterine carcinosarcoma is worse than that of endometrial carcinoma with adjustment for known adverse prognostic factors [25, 72]. The behaviour of uterine carcinosarcoma has been likened to that of dedifferentiated endometrial carcinoma [72]. This poor prognosis compared to other uterine malignancies is primarily attributed to the high rates of distant metastases and early recurrences often attributed to the advanced stage at initial clinical presentation [17].

Prognostic features though well-studied in the literature are reported with conflicting results. The most important prognostic factor is the extent of tumour at initial presentation, as extrauterine spread is associated with very poor survival outcomes [14]. Older patients (over 70 years of age) have been reported to have a poorer outcome than the young, which may be attributed to preexisting comorbid factors such as a poorer performance status resulting in less aggressive therapy [30]; however, other literature does not support this finding [29]. Oral contraceptives are protective against uterine carcinosarcomas [25] whereas tamoxifen increases the relative risk fourfold [33]. Preoperative levels of CA125 are correlated with extrauterine disease and increased myometrial invasion. Postoperative increases of CA125 have been reported as a significant independent prognostic factor for death [32]. 

Tumour characteristics such as myometrial invasion of less than one-third of the uterus with no detectable metastasis, and a size less than 7 cm are all associated with a favourable outcome in some reports [73]. Most studies agree that deeper myometrial invasion increases the risk of extrauterine extension [30] and on multivariant analysis stage has been reported as the most important prognostic factor [55] and predictor of patient outcome [74]. Other studies have not found the initial tumour size to significantly alter survival rates [30]. Specifically in early-stage uterine carcinosarcomas, additional prognostic factors associated with a worse outcome include lymphovascular space involvement, the histology of the carcinomatous component, the extent of the sarcomatous component, and the presence of heterologous elements [74]. Homologous-type uterine carcinosarcoma confers a better prognosis than the heterologous-type in some studies [30], but this relationship is not supported by others [29]. Positive peritoneal cytology is associated with poor prognosis in uterine carcinosarcoma [10]. In some studies, serous or clear cell carcinoma as the epithelial element is associated with poorer survival outcomes [32]. Tumour characteristics of molecular markers such as expression of p53 in older women are associated with a shorter mean survival, while p53 negative tumours occurring in younger women have a longer survival [24, 38]. Immunohistochemical tumour expression of other cell cycle and apoptotic regulatory proteins such as p16 and Mcl-1 are also associated with longer survivals [24, 38, 71]. Trends observed in such individual series are difficult to generalize due to small sample sizes and need to be validated as predictive and/or prognostic markers with further research in larger tumour populations.

## 11. Conclusions

**Figure 5:**
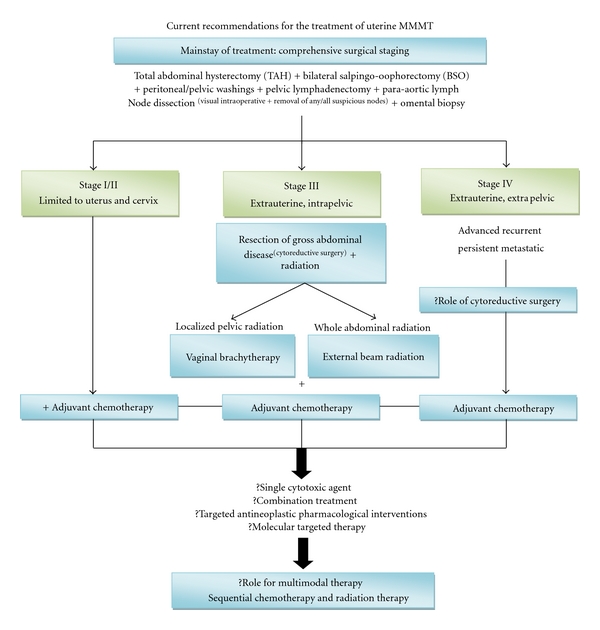
This flow chart summarizes the current recommendations for the treatment of uterine carcinosarcomas. Due to the rapidly progressive nature of this neoplasm, we recommend adjuvant chemotherapy even in Stage I/II lesions as we believe that the late clinical presentation of these cases is usually associated with silent microscopic disseminated disease. Uncertainties regarding the value of aggressive cytoreductive surgery in Stage IV disease are questionable and indicated with “?”. Similarly, the most effective protocol for chemotherapy is also unknown and indicated by “?”. Likewise, the role for multimodal therapy in this neoplasm is yet to be determined (?).

Uterine carcinosarcoma is a rare, highly aggressive, rapidly progressing neoplasm associated with a poor prognosis that has not significantly improved in the past thirty years despite advances in imaging and adjuvant therapies. Controversies continue to linger in many areas of uterine carcinosarcoma, as summarized in Table 2. The optimal management modality remains controversial, with discrepancies regarding patient outcome to lymphadenectomy and radiation therapy. Additionally, various chemotherapeutic protocols have been attempted with varying results. There are no current consensus guidelines for the management of this rare disease. The rarity of this neoplasm resulting in small sample size has precluded large trials for evaluation of various treatment protocols. Yet, uterine carcinosarcoma though rare needs to be recognized as a distinct entity, as it is highly aggressive. To maximize the probability of cure with improved survival outcomes the future of uterine carcinosarcoma management is to develop consensus guidelines of treatment. This can be realized by prospective multicentric, multi-institutional collaborative randomized trials of treatment protocols with novel multimodality strategies that include a multidisciplinary approach of surgery, radiotherapy, and potentially evolving specific systemic therapy with targeted antineoplastic pharmacological interventions. In summary, the current proposed recommendation for the management of uterine carcinosarcoma is outlined in Figure 5.

## Figures and Tables

**Table 1:** Typical patient profile presenting with uterine carcinosarcoma.

(i) Elderly female (usually 60–70 years), usually postmenopausal
(ii) Presents with pyometra with vagina bleeding, bloody/watery discharge, abdominal pain, and/or mass
(iii) Often past history of tamoxifen use
(iv) May be obese, hypertensive, nulliparous, and/or diabetic
(v) No previous history of uterine problems

**Table 2:** Controversies in uterine carcinosarcoma.

*(i) Origins: *sarcomatous versus carcinomatous monoclonal versus biclonal versus polyclonal.
*(ii) Demographics*: more common in Afro-American versus Caucasian women.
*(iii) Aetiology: *radiation inducible tumour versus metaplastic versus dedifferentiation versus common stem cell.
*(iv) Pathogenesis: *collision theory versus combination theory versus conversion theory.
*(v) Risk factors: * beneficial effect of oral contraceptives versus detrimental effect of exogenous estrogens.
*(vi) Presentation:* symptomatic (pyometra/vaginal bleeding/abdominal pain) versus asymptomatic.
*(vii) Microscopic:* biphasic components—separated versus merged.
*(viii) MRI description:* endophytic with architectural obliteration versus exophytic with no invasiveness.
*(ix) Sonography: * diagnostic use—yes versus no technique—transabdominal versus transvaginal.
*(x) Surgery: *lymphadenectomy versus nolymph-node dissection.
*(xi) Adjuvant therapy:* radiotherapy versus chemotherapy versus molecular targeted versus multimodality therapy.
*(xii) Radiotherapy: *locoregional control versus improved overall survival limited pelvic radiation versus whole abdominal radiation.
*(xiii) Chemotherapy:* single-agent versus combination versus targeted antineoplastic therapy.
*(xiv) Prognostic features: *?significance of tumour size, patient age, and histology of sarcomatous element.

## References

[1] N’Kanza AL, Jobanputra S, Farmer P, Lovecchio J, Yelon JA, Rudloff U (2005). Central nervous system involvement from malignant mixed Mullerian tumor (MMMT) of the uterus. *Archives of Gynecology and Obstetrics*.

[2] Banik T, Halder D, Gupta N, Dey P Malignant mixed Mullerian tumor of the uterus: diagnosis of a case by fine-needle aspiration cytology and review of literature.

[3] Ahuja A, Safaya R, Prakash G, Kumar L, Shukla NK (2011). Primary mixed mullerian tumor of the vagina—a case report with review of the literature. *Pathology Research and Practice*.

[4] Sharma NK, Sorosky JI, Bender D, Fletcher MS, Sood AK (2005). Malignant mixed mullerian tumor (MMMT) of the cervix. *Gynecologic Oncology*.

[5] Duman BB, Kara IO, Gunaldi M, Ercolak V (2011). Malignant mixed Mullerian tumor of the ovary with two cases and review of the literature. *Archives of Gynecology and Obstetrics*.

[6] Shen YM, Xie YP, Xu L (2010). Malignant mixed mullerian tumor of the fallopian tube: report of two cases and review of literature. *Archives of Gynecology and Obstetrics*.

[7] El-Nashar SA, Mariani A (2011). Uterine carcinosarcoma. *Clinical Obstetrics and Gynecology*.

[8] Bansal N, Herzog TJ, Seshan VE (2008). Uterine carcinosarcomas and grade 3 endometrioid cancers: evidence for distinct tumor behavior. *Obstetrics and Gynecology*.

[9] Clayton Smith DC, Kenneth Macdonald O, Gaffney DK (2008). The impact of adjuvant radiation therapy on survival in women with uterine carcinosarcoma. *Radiotherapy and Oncology*.

[10] Garg G, Kruger M, Christensen C, Deppe G, Toy EP Stage III uterine carcinosarcoma: 2009 international federation of gynecology and obstetrics staging system and prognostic determinants.

[11] Garg G, Shah JP, Kumar S, Bryant CS, Munkarah A, Morris RT (2010). Ovarian and uterine carcinosarcomas: a comparative analysis of prognostic variables and survival outcomes. *International Journal of Gynecological Cancer*.

[12] Nemani D, Mitra N, Guo M, Lin L (2008). Assessing the effects of lymphadenectomy and radiation therapy in patients with uterine carcinosarcoma: a SEER analysis. *Gynecologic Oncology*.

[13] Wright JD, Seshan VE, Shah M (2008). The role of radiation in improving survival for early-stage carcinosarcoma and leiomyosarcoma. *American Journal of Obstetrics and Gynecology*.

[14] Bosquet JS, Terstriep SA, Cliby WA (2010). The impact of multi-modal therapy on survival for uterine carcinosarcomas. *Gynecologic Oncology*.

[15] Tanaka YO, Tsunoda H, Minami R, Yoshikawa H, Minami M (2008). Carcinosarcoma of the uterus: MR findings. *Journal of Magnetic Resonance Imaging*.

[16] Kernochan LE, Garcia RL (2009). Carcinosarcomas (malignant mixed mullerian tumor) of the uterus: advances in elucidation of biologic and clinical characteristics. *Journal of the National Comprehensive Cancer Network*.

[17] Ramondetta LM, Burke TW, Jhingran A (2003). A phase II trial of cisplatin, ifosfamide, and mesna in patients with advanced or recurrent uterine malignant mixed müllerian tumors with evaluation of potential molecular targets. *Gynecologic Oncology*.

[18] Sutton G, Kauderer J, Carson LF, Lentz SS, Whitney CW, Gallion H (2005). Adjuvant ifosfamide and cisplatin in patients with completely resected stage I or II carcinosarcomas (mixed mesodermal tumors) of the uterus: a Gynecologic Oncology Group study. *Gynecologic Oncology*.

[19] de Jong RA, Nijman HW, Wijbrandi TF, Reyners AK, Boezen HM, Hollema H Molecular markers and clinical behavior of uterine carcinosarcomas: focus on the epithelial tumor component.

[20] Arora P, Rao S, Khurana N, Talwar D, Tanwar R (2011). Malignant mixed Mullerian tumor of broad ligament with synchronous ovarian and endometrial carcinoma: a rare association. *Journal of Cancer Research and Therapeutics*.

[21] Kloos I, Delaloge S, Pautier P (2002). Tamoxifen-related uterine carcinosarcomas occur under/after prolonged treatment: report of five cases and review of the literature. *International Journal of Gynecological Cancer*.

[22] Jin Z, Ogata S, Tamura G (2003). Carcinosarcomas (malignant mullerian mixed tumors) of the uterus and ovary: a genetic study with special reference to histogenesis. *International Journal of Gynecological Pathology*.

[23] Kuyumcuoğlu U, Kale A (2009). Homologous type of malignant mixed Mullerian tumor of the uterus presenting as a cervical mass. *Journal of the Chinese Medical Association*.

[24] Buza N, Tavassoli FA (2009). Comparative analysis of P16 and P53 expression in uterine malignant mixed mullerian tumors. *International Journal of Gynecological Pathology*.

[25] McCluggage WG (2002). Uterine carcinosarcomas (malignant mixed Mullerian tumors) are metaplastic carcinomas. *International Journal of Gynecological Cancer*.

[26] Meredith RF, Eisert DR, Kaka Z, Hodgson SE, Johnston GA, Boutselis JG (1986). An excess of uterine sarcomas after pelvic irradiation. *Cancer*.

[27] Doss LL, Llorens AS, Henriquez EM (1984). Carcinosarcoma of the uterus: a 40-year experience from the state of Missouri. *Gynecologic Oncology*.

[28] Callister M, Ramondetta LM, Jhingran A, Burke TW, Eifel PJ (2004). Malignant mixed mullerian tumors of the uterus: analysis of patterns of failure, prognostic factors, and treatment outcome. *International Journal of Radiation Oncology Biology Physics*.

[29] Niculescu M, Simionescu C, Novac L, Mogoanta L, Stanescu RM (2007). The uterine carcinosarcoma—a case report. *Romanian Journal of Morphology and Embryology*.

[30] Inthasorn P, Carter J, Valmadre S, Beale P, Russell P, Dalrymple C (2002). Analysis of clinicopathologic factors in malignant mixed Mullerian tumors of the uterine corpus. *International Journal of Gynecological Cancer*.

[31] Ho KC, Lai CH, Wu TI (2008). ^18^F-fluorodeoxyglucose positron emission tomography in uterine carcinosarcoma. *European Journal of Nuclear Medicine and Molecular Imaging*.

[32] Huang GS, Chiu LG, Gebb JS (2007). Serum CA125 predicts extrauterine disease and survival in uterine carcinosarcoma. *Gynecologic Oncology*.

[33] Kajo K, Zubor P, Spacek J, Ryska A (2007). Carcinosarcoma of the uterus with melanocytic differentiation. *Pathology Research and Practice*.

[34] Temkin SM, Hellmann M, Lee YC, Abulafia O (2007). Early-stage carcinosarcoma of the uterus: the significance of lymph node count. *International Journal of Gynecological Cancer*.

[35] Ohguri T, Aoki T, Watanabe H (2002). MRI findings including gadolinium-enhanced dynamic studies of malignant, mixed mesodermal tumors of the uterus: differentiation from endometrial carcinomas. *European Radiology*.

[36] Genever AV, Abdi S (2011). Can MRI predict the diagnosis of endometrial carcinosarcoma?. *Clinical Radiology*.

[37] Brown L (2008). Pathology of uterine malignancies. *Clinical Oncology*.

[38] Kanthan R, Senger JLB, Diudea D (2010). Malignant mixed Mullerian tumors of the uterus: histopathological evaluation of cell cycle and apoptotic regulatory proteins. *World Journal of Surgical Oncology*.

[39] Livasy CA, Reading FC, Moore DT, Boggess JF, Lininger RA (2006). EGFR expression and HER2/neu overexpression/amplification in endometrial carcinosarcoma. *Gynecologic Oncology*.

[40] Raspollini MR, Susini T, Amunni G (2005). COX-2, c-KIT and HER-2/neu expression in uterine carcinosarcomas: prognostic factors or potential markers for targeted therapies?. *Gynecologic Oncology*.

[41] Sawada M, Tsuda H, Kimura M (2003). Different expression patterns of KIT, EGFR, and HER-2 (c-erbB-2) oncoproteins between epithelial and mesenchymal components in uterine carcinosarcoma. *Cancer Science*.

[42] Swisher EM, Gown AM, Skelly M (1996). The expression of epidermal growth factor receptor, HER-2/Neu, p53, and Ki-67 antigen in uterine malignant mixed mesodermal tumors and adenosarcoma. *Gynecologic Oncology*.

[43] Worthington JL, Balfe DM, Lee JK (1986). Uterine neoplasms: MR imaging. *Radiology*.

[44] Shapeero LG, Hricak H (1989). Mixed mullerian sarcoma of the uterus: MR imaging findings. *American Journal of Roentgenology*.

[45] Bharwani N, Newland A, Tunariu N (2010). MRI appearances of uterine malignant mixed mullerian tumors. *American Journal of Roentgenology*.

[46] Smith T, Moy L, Runowicz C (1997). Mullerian mixed tumors: CT characteristics with clinical and pathologic observations. *American Journal of Roentgenology*.

[47] Teo SY, Babagbemi KT, Peters HE, Mortele KJ (2008). Primary malignant mixed Mullerian tumor of the uterus: findings on sonography, CT, and gadolinium-enhanced MRI. *American Journal of Roentgenology*.

[48] Umesaki N, Tanaka T, Miyama M, Ogita S, Ochi H (2000). Combined diagnostic imaging of uterine carcinosarcoma: a case report. *International Journal of Gynecological Cancer*.

[49] Umesaki N, Tanaka T, Miyama M (2001). Positron emission tomography with ^18^F-fluorodeoxyglucose of uterine sarcoma: a comparison with magnetic resonance imaging and power Doppler imaging. *Gynecologic Oncology*.

[50] Murakami M, Tsukada H, Shida M (2006). Whole-body positron emission tomography with F-18 fluorodeoxyglucose for the detection of recurrence in uterine sarcomas. *International Journal of Gynecological Cancer*.

[51] Markman M (2004). Chemotherapeutic management of recurrent/metastatic uterine carcinosarcomas (malignant mixed mullerian tumors): time for a re-appraisal?. *Journal of Cancer Research and Clinical Oncology*.

[52] Lacour RA, Euscher E, Atkinson EN (2011). A phase II trial of paclitaxel and carboplatin in women with advanced or recurrent uterine carcinosarcoma. *International Journal of Gynecological Cancer*.

[53] Ulbricht LJ, Kunert M, Gremmler B, Evagelopoulos N, Krian A, Moege J (2009). Intracardiac metastasis of a Malignant Mixed Mullerian Tumor (MMMT): progressive dyspnoea due to obstruction of the left atrium and the left ventricle without left ventricular dysfunction or primary lung disease. *Wiener Medizinische Wochenschrift*.

[54] Menczer J, Levy T, Piura B (2005). A comparison between different postoperative treatment modalities of uterine carcinosarcoma. *Gynecologic Oncology*.

[55] Vorgias G, Fotiou S (2010). The role of lymphadenectomy in uterine carcinosarcomas (malignant mixed mullerian tumours): a critical literature review. *Archives of Gynecology and Obstetrics*.

[56] Ozguroglu M, Bilici A, Ilvan S (2008). Determining predominating histologic component in malignant mixed mullerian tumors: is it worth it?. *International Journal of Gynecological Cancer*.

[57] Dusenbery KE, Potish RA, Argenta PA, Judson PL (2005). On the apparent failure of adjuvant pelvic radiotherapy to improve survival for women with uterine sarcomas confined to the uterus. *American Journal of Clinical Oncology*.

[58] Sartori E, Bazzurini L, Gadducci A (1997). Carcinosarcoma of the uterus: a clinicopathological multicenter CTF study. *Gynecologic Oncology*.

[59] Wolfson AH, Brady MF, Rocereto T (2007). A gynecologic oncology group randomized phase III trial of whole abdominal irradiation (WAI) vs. cisplatin-ifosfamide and mesna (CIM) as post-surgical therapy in stage I-IV carcinosarcoma (CS) of the uterus. *Gynecologic Oncology*.

[60] Hoskins PJ, Le N, Ellard S (2008). Carboplatin plus paclitaxel for advanced or recurrent uterine malignant mixed mullerian tumors. The British Columbia Cancer Agency experience. *Gynecologic Oncology*.

[61] Rose PG, Piver MS, Tsukada Y, Lau T (1989). Patterns of metastasis in uterine sarcoma. An autopsy study. *Cancer*.

[62] Powell MA, Filiaci VL, Rose PG (2010). Phase II evaluation of paclitaxel and carboplatin in the treatment of carcinosarcoma of the uterus: a Gynecologic Oncology Group study. *Journal of Clinical Oncology*.

[63] Miller DS, Blessing JA, Schilder J, Munkarah A, Lee YC (2005). Phase II evaluation of topotecan in carcinosarcoma of the uterus: a Gynecologic Oncology Group study. *Gynecologic Oncology*.

[64] Nimeiri HS, Oza AM, Morgan RJ (2010). A phase II study of sorafenib in advanced uterine carcinoma/carcinosarcoma: a trial of the Chicago, PMH, and California Phase II Consortia. *Gynecologic Oncology*.

[65] Huh WK, Sill MW, Darcy KM (2010). Efficacy and safety of imatinib mesylate (Gleevec) and immunohistochemical expression of c-Kit and PDGFR-*β* in a Gynecologic Oncology Group Phase Il Trial in women with recurrent or persistent carcinosarcomas of the uterus. *Gynecologic Oncology*.

[66] Crotzer DR, Wolf JK, Gano JB, Gershenson DM, Levenback C (2007). A pilot study of cisplatin, ifosfamide and mesna in the treatment of malignant mixed mesodermal tumors of the ovary. *Gynecologic Oncology*.

[67] Sutton G, Brunetto VL, Kilgore L (2000). A phase III trial of ifosfamide with or without cisplatin in carcinosarcoma of the uterus: a Gynecologic Oncology Group study. *Gynecologic Oncology*.

[68] Homesley HD, Filiaci V, Markman M (2007). Phase III trial of ifosfamide with or without paclitaxel in advanced uterine carcinosarcoma: a gynecologic oncology group study. *Journal of Clinical Oncology*.

[69] Miller BE, Blessing JA, Stehman FB (2010). A phase II evaluation of weekly gemcitabine and docetaxel for second-line treatment of recurrent carcinosarcoma of the uterus: a gynecologic oncology group study. *Gynecologic Oncology*.

[70] Bland AE, Stone R, Heuser C (2009). A clinical and biological comparison between malignant mixed mullerian tumors and grade 3 endometrioid endometrial carcinomas. *International Journal of Gynecological Cancer*.

[71] Robinson-Bennett B, Belch RZ, Han AC (2006). Loss of p16 in recurrent malignant mixed mullerian tumors of the uterus. *International Journal of Gynecological Cancer*.

[72] Vaidya AP, Horowitz NS, Oliva E, Halpern EF, Duska LR (2006). Uterine malignant mixed mullerian tumors should not be included in studies of endometrial carcinoma. *Gynecologic Oncology*.

[73] Magnani KK, Dubey S, Rai S (2010). Malignant mixed Müllerian tumor of the uterus associated with tamoxifen therapy for breast cancer. *Indian Journal of Pathology and Microbiology*.

[74] Ferguson SE, Tornos C, Hummer A, Barakat RR, Soslow RA (2007). Prognostic features of surgical stage I uterine carcinosarcoma. *American Journal of Surgical Pathology*.

